# Semi-Biosynthetic Production of Surface-Binding Adhesive Antimicrobial Peptides Using Intein-Mediated Protein Ligation

**DOI:** 10.3390/ijms232315202

**Published:** 2022-12-02

**Authors:** Young Eun Hwang, Seonghun Im, Ju Hyun Cho, Wonsik Lee, Byung-Kwan Cho, Bong Hyun Sung, Sun Chang Kim

**Affiliations:** 1 Department of Biological Sciences, Korea Advanced Institute of Science and Technology, Daejeon 34141, Republic of Korea; 2 Synthetic Biology and Bioengineering Research Center, Korea Research Institute of Bioscience and Biotechnology, Daejeon 34141, Republic of Korea; 3 Center for Industrialization of Agricultural and Livestock Microorganisms (CIALM), Jeongeup 56212, Republic of Korea; 4 Division of Applied Life Science (BK21Four), Research Institute of Life Sciences, Gyeongsang National University, Jinju 52828, Republic of Korea; 5 School of Pharmacy, Sungkyunkwan University, Suwon 16419, Republic of Korea

**Keywords:** antimicrobial peptide, *Escherichia coli*, intein, intein-mediated protein ligation

## Abstract

Microbial infections remain a global health concern, calling for the urgent need to implement effective prevention measures. Antimicrobial peptides (AMPs) have been extensively studied as potential antimicrobial coating agents. However, an efficient and economical method for AMP production is lacking. Here, we synthesized the direct coating adhesive AMP, NKC-DOPA_5_, composed of NKC, a potent AMP, and repeats of the adhesive amino acid 3,4-dihydroxyphenylalanine (DOPA) via an intein-mediated protein ligation strategy. NKC was expressed as a soluble fusion protein His-NKC-GyrA (HNG) in *Escherichia coli*, comprising an N-terminal 6× His-tag and a C-terminal *Mxe* GyrA intein. The HNG protein was efficiently produced in a 500-L fermenter, with a titer of 1.63 g/L. The NKC-thioester was released from the purified HNG fusion protein by thiol attack and subsequently ligated with chemically synthesized Cys-DOPA_5_. The ligated peptide His-NKC-Cys-DOPA_5_ was obtained at a yield of 88.7%. The purified His-NKC-Cys-DOPA_5_ possessed surface-binding and antimicrobial properties identical to those of the peptide obtained via solid-phase peptide synthesis. His-NKC-Cys-DOPA_5_ can be applied as a practical and functional antimicrobial coating to various materials, such as medical devices and home appliances.

## 1. Introduction

Antimicrobial peptides (AMPs), small proteins with a molecular weight of <10 kDa produced by all organisms as the first line of defense against external damage or infection, are mostly cationic peptides with amphipathic properties associated with non-specific and rapid antimicrobial mechanisms against a wide range of microorganisms, including Gram-positive and Gram-negative bacteria, fungi, and certain viruses [1,2,3,4,5]. The emergence of multidrug-resistant bacteria has rendered conventional antibiotics with a single killing mechanism ineffective. In contrast, AMPs have multiple low-affinity actions and a negligible propensity to trigger resistance and are also effective in killing drug-resistant bacteria [6,7].

Because of these advantages, AMPs have been proposed as infection-resistant coating agents to combat microbial infections, which are a leading cause of death worldwide. The strategies used for AMP coating include the covalent attachment of AMPs [8,9,10,11], the use of chimeric peptides comprising antimicrobial motifs and specific binding peptides, such as hydroxyapatite-binding peptide [12], titanium-binding peptide [13,14,15], or polystyrene-binding peptide [16], and catechol chemistry [17,18,19,20]. Catechol groups enable strong, substrate-independent adhesion in mild conditions and hence are attractive candidates for immobilizing AMP on various surfaces. Among the various catechol-containing compounds, 3,4-dihydroxyphenylalanine (DOPA), derived from the posttranslational oxidation of tyrosine (Tyr) and a key molecule in mussel adhesive proteins, endows adhesive ability when incorporated into AMP sequences, thereby allowing for one-step coating without chemical activation. Therefore, DOPA-incorporated AMPs have application potential as single-molecule antimicrobial coating agents for various industries, including medical devices and home appliances [21,22,23].

However, the lack of a cost-effective production system hinders their application. Although recombinant DNA technology is a cost-effective strategy to produce AMPs, various protein expression systems are limited in their ability to produce proteins with unnatural amino acids (UAAs), such as DOPA. Current strategies for the biosynthesis of UAA-containing proteins are reassigning codons for UAAs and utilizing orthogonal translation machinery, including complementary sets of aminoacyl-tRNA (aa-tRNA) and aminoacyl-tRNA synthetase (aaRS) [24,25]. An engineered orthogonal aa-tRNA–aaRS pair can incorporate DOPA, although specifically incorporating DOPA without Tyr may be challenging owing to their structural similarity [26]. DOPA also can be incorporated into proteins via the enzymatic conversion of Tyr; however, unwanted sequential DOPA oxidation to DOPA-quinone due to the diphenolase activity of tyrosinase can occur and is difficult to control [27]. In addition, there are two challenges to overcome to establish AMP expression in *Escherichia coli*: first, production host cells are susceptible to AMPs due to their highly positive charge, and second, AMPs are prone to proteolytic degradation due to their low molecular weight [28,29]. To reduce toxicity to the host strain, AMPs can be expressed as fusion proteins [30,31].

To produce UAA-containing AMPs, a semi-biosynthetic method that combines the biological production of AMPs as fusion proteins and the chemical production of UAAs can overcome the limitations of the biosynthesis of unnatural protein molecules. The concept of native chemical ligation, developed by Dawson in 1994, has been applied for conjugating two fragments (one with a C-terminal thioester and another with an N-terminal cysteine) that will be chemoselectively ligated via a peptide bond to either synthesize a larger protein or introduce functional moieties [32]. This concept has been expanded to develop an expressed protein ligation method in which an intein protein capable of producing a thioester at the C-terminus of a recombinant protein, which can be ligated with a synthetic peptide segment to synthesize functionalized proteins with minimal or no ligation scar [33,34].

Herein, we propose an effective ligation-based method to produce AMPs with UAAs, which are difficult to produce biologically. To the best of our knowledge, this is the first study to attempt the semi-biosynthetic production of AMPs with UAAs using intein-mediated protein ligation. The reported method was successfully applied to the synthesis of a potent adhesive peptide harboring the non-canonical amino acid DOPA.

## 2. Results and Discussion

### 2.1. Semi-Synthesis of Adhesive AMP Containing DOPA

**Figure 1:**
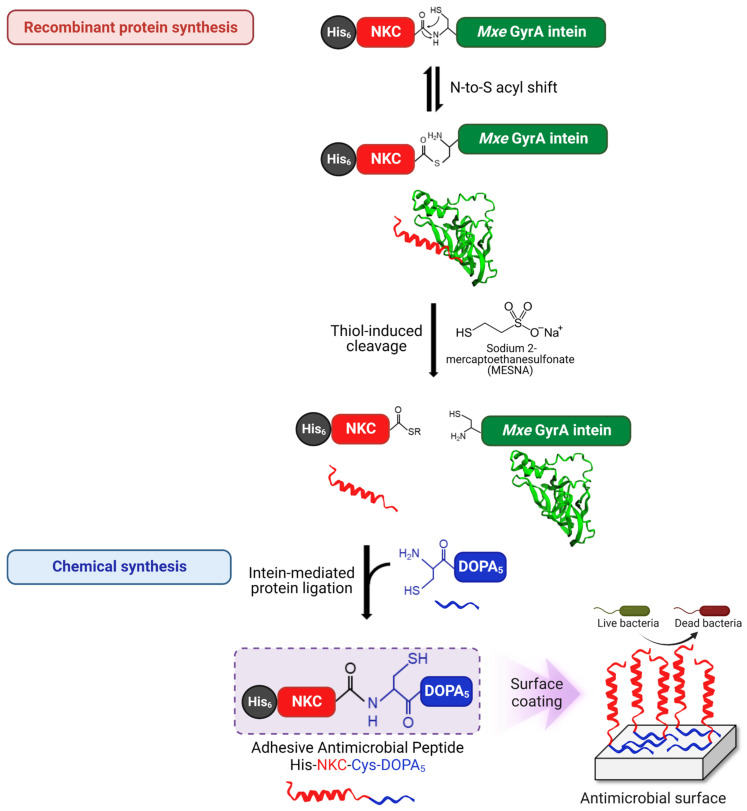
Semi-synthesis of antimicrobial peptides using an intein-mediated protein ligation strategy. The His_6_-tag was used for affinity purification, while *Mxe* GyrA intein was fused to the C-terminus of NKC. The thiol-mediated cleavage of the intein fusion protein using sodium 2-mercaptoethanesulfonate (MESNA) yielded the target peptide with a reactive thioester. The resulting NKC-thioester was mixed with DOPA_5_ with an N-terminal cysteine, leading to spontaneous peptide bond formation between the recombinantly obtained NKC-thioester and the chemically synthesized Cys-DOPA_5_ via a nucleophilic attack of the N-terminal cysteine on the NKC-thioester.

We have previously reported a potent adhesive AMP, NKC-DOPA_5_, as a potential antimicrobial coating agent [35]. The peptide, comprising NKC (APKAMKLLKKLLKLQKKGI) as an antimicrobial domain [36,37] and five DOPA residues as an adhesion domain, was successfully immobilized onto the surface of various materials via one-step coating without surface pretreatment and showed strong antimicrobial activity and stability. Several excellent adhesive AMPs have been developed; however, owing to the lack of an efficient production method, their use has been limited [38]. In this study, a semi-synthetic approach combining the advantages of biological and chemical methods was employed to produce an AMP containing DOPA. Concretely, the antimicrobial domain (NKC and flexible linker) fused with intein was produced biologically, and the adhesion domain (DOPA_5_) was synthesized chemically, and these two were linked via intein-mediated ligation (Figure 1). The expression cassette, His-NKC-GyrA (HNG), comprised six histidine (His) residues for protein purification, the potent AMP NKC, and a non-self-cleaving *Mycobacterium xenopi* DNA gyrase A (*Mxe* GyrA) intein. The amino acid sequences of the HNG fusion proteins are listed in Table 1. For the efficient semi-synthesis of the peptide, the novel adhesive peptide His-NKC-Cys-DOPA_5_ was prepared with a slightly different sequence from NKC-DOPA_5_; a glycine residue (underscored in Table 1) was added preceding the cleavage site for efficient cleavage by GyrA intein, following the manufacturer’s instruction. A cysteine residue was added between the linker and the DOPA moieties for native chemical ligation; however, the overall structure was similar, with the same minimal inhibitory concentration (MIC) value of 16 μM, suggesting identical antibacterial activity (Table 1).

### 2.2. Expression and Purification of HNG Fusion Proteins

**Figure 2:**
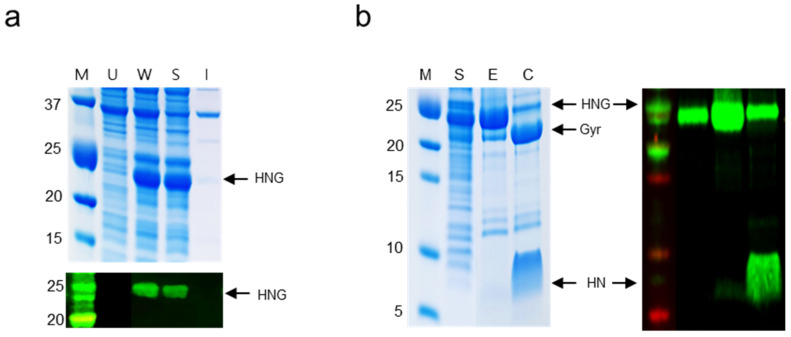
Expression of the His-NKC-GyrA (HNG) fusion protein. (**a**) SDS-PAGE and Western blot analyses (anti-His) of HNG expression. Lane M: protein molecular weight marker; Lane U: uninduced *E. coli* lysate; Lane W: whole cell lysate; Lane S: soluble fraction; Lane I: insoluble fraction. The target protein is indicated by arrows. (**b**) Analysis of purification of the HNG protein and cleavage activity of GyrA intein. Lane M: protein molecular weight marker; Lane S: soluble fraction; Lane E: elution fraction with 250 mM imidazole; Lane C: product of intein cleavage induced by MESNA. Gyr and HN indicate GyrA intein and His-NKC-thioester, respectively. The green bands on the black background represent the results of Western blot analysis.

The HNG fragment was cloned into the bacterial expression vector pET21b, designated pET21b-HNG. The plasmid was transformed into the bacterial expression host, *E. coli* BL21 (DE3). The expression of the HNG fusion protein was induced with 0.1 mM isopropyl-ß-D-1-thiogalactopyranoside (IPTG) for 16 h. The protein expression of HNG, approximately 24.6 kDa in size, was confirmed using sodium dodecyl sulfate-polyacrylamide gel electrophoresis (SDS-PAGE) (Figure 2a). The HNG protein was purified using nickel affinity chromatography; the eluted sample was separated on an SDS-PAGE gel stained with Coomassie Blue staining solution (Figure 2b). The purified proteins were further subjected to ultrafiltration using a polyethersulfone (PES) membrane (molecular weight cut-off: 5 kDa). Approximately 483.6 mg of pure recombinant HNG was obtained from 1 L of *E. coli* culture. The cleavage activity of the purified HNG protein was confirmed by incubation with 100 mM sodium 2-mercaptoethanesulfonate (MESNA) at 25 °C for 24 h. The HNG protein was split into GyrA and His-NKC-thioester.

### 2.3. Fed-Batch Fermentation

**Figure 3:**
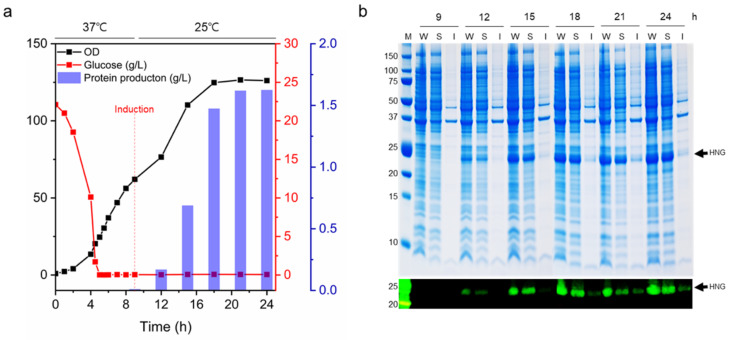
Fed-batch fermentation of *E. coli* BL21 (DE3)-pET21b-HNG. (**a**) *E. coli* fed-batch fermentation kinetics for the production of HNG. Cells were cultivated in batch mode until the initially present glucose was depleted. After 5 h, glucose feeding was initiated at a predefined rate. At an OD_600_ of 62, protein expression was induced with 0.1 mM IPTG. Samples were collected to monitor cell OD_600_ (black) and medium glucose content (red). (**b**) Culture samples collected at induction times of 9, 12, 15, 18, 21, and 24 h were analyzed using SDS-PAGE and Western blotting with an anti-His antibody. Lane M: protein molecular weight marker; Lane W: whole cell lysate; Lane S: soluble fraction; Lane I: insoluble fraction. The target protein is indicated by arrows.

For the large-scale production of the HNG fusion protein, *E. coli* BL21 (DE3) harboring pET21b-HNG was cultivated in a 500-L fermenter. Feeding medium was continuously added after the initial glucose was depleted after 5 h of cultivation. The feeding rate was adjusted based on glucose consumption. When the optical density at 600 nm (OD_600_) reached 62 around 9 h post-inoculation, the cultivation temperature was reduced from 37 °C to 25 °C, and HNG fusion protein expression in the recombinant *E. coli* was induced with 0.1 mM IPTG for 15 h. Three hours following IPTG induction, HNG protein production began (Figure 3a). The final volume following fermentation was approximately 255 L. The maximum cell density was OD_600_ = 126, and the dry cell weight after lyophilization was approximately 45.2 g/L. The target protein titer determined using a His-Tag Detection ELISA kit was 1.63 g/L (28.1 mg/g dry cell weight). As shown in Figure 3b, protein bands of approximately 24.6 kDa, corresponding to the molecular weight of HNG, were observed on the SDS-PAGE gel (indicated by arrows) following induction. HNG protein production increased with incubation time.

### 2.4. Intein-Mediated Cleavage and Protein Ligation

**Figure 4:**
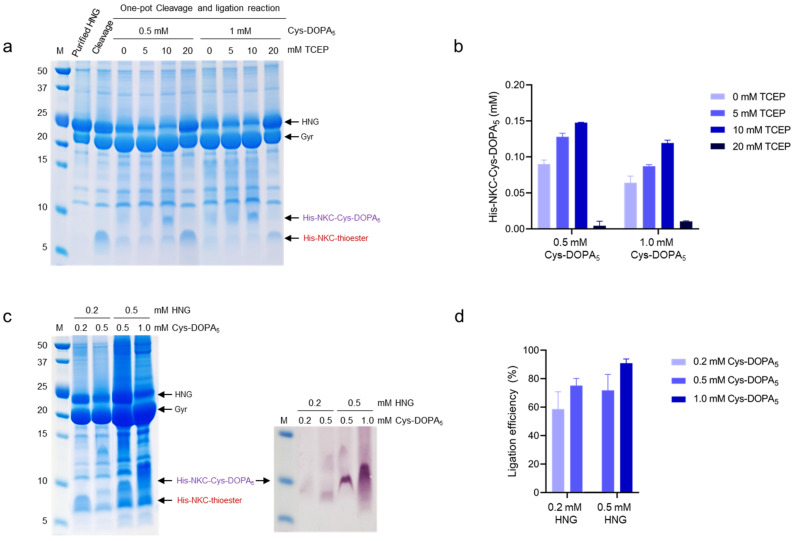
One-pot intein-mediated cleavage and peptide ligation. (**a**) SDS-PAGE analysis of intein-mediated protein ligation according to TCEP concentration. The reactions were performed using the indicated Cys-DOPA_5_ and TCEP concentrations to 0.2 mM HNG. (**b**) Quantification of the ligated peptide using RP-HPLC (detection at 280 nm). (**c**) Analysis of intein-mediated protein ligation on an SDS-PAGE gel stained with Coomassie Blue and nitroblue tetrazolium (NBT; purple bands). The reactions were performed at the indicated HNG and Cys-DOPA_5_ concentrations. The ligation products show positive NBT staining, indicating the presence of active DOPA. (**d**) Quantification of the ligated peptide using RP-HPLC (detection at 280 nm).

Intein-mediated cleavage was induced by adding the thiol nucleophile MESNA, which released the target peptide (NKC) from the GyrA intein while simultaneously producing a carboxy-terminal thioester intermediate on NKC. Subsequently, Cys-DOPA_5_ was covalently attached to the NKC-thioester to form a new peptide bond (Figure 1). To optimize the cleavage and ligation conditions, 0.5 mM and 1 mM Cys-DOPA_5_ peptide were added to 0.2 mM HNG protein with 100 mM MESNA and different concentrations of the sulfhydryl-free reducing agent Tris(2-carboxyethyl)phosphine (TCEP). TCEP maintains a highly reduced environment, thereby preventing unwanted cysteine oxidation and improving ligation efficiency [39,40]. To determine the optimal concentration of TCEP in the ligation reaction, TCEP was added at 0, 5, 10, or 20 mM. The ligation products were analyzed using SDS-PAGE coupled with reversed-phase high-performance liquid chromatography (RP-HPLC). Upon intein cleavage, His-NKC-thioester (approximately 7 kDa) cleaved from the HNG protein (24.6 kDa in size) appeared (Figure 4a). Following ligation, an additional peptide band (approximately 9 kDa) appeared, suggesting the successful ligation of His-NKC-thioester and Cys-DOPA_5_. Cys-DOPA_5_ (1.017 kDa in size) was not detected by SDS-PAGE because of the low molecular weight. The production of His-NKC-Cys-DOPA_5_ increased as the TCEP concentration increased from 0 to 10 mM; however, at 20 mM, it decreased (Figure 4b). The optimal TCEP concentration for maximum ligation efficiency was determined to be 10 mM, which is the concentration used for intein-mediated protein ligation in previous studies [40,41]; therefore, we employed a ligation buffer containing 10 mM TCEP in all further experiments. In addition, His-NKC-Cys-DOPA_5_ production was higher at an HNG-to-Cys-DOPA_5_ molar ratio of 1:2.5 (0.2 mM HNG and 0.5 mM Cys-DOPA_5_) than at a ratio of 1:5 (0.2 mM HNG and 1.0 mM Cys-DOPA_5_); hence, a higher molar ratio of Cys-DOPA_5_ to HNG was not required. Purified HNG protein can be cleaved by autolysis. However, since the resulting His-NKC-thioester was not visible on the SDS-PAGE, the amount of autolysis was judged to be very small. The band at the GyrA position shown in the control lane in Figure 4a was a non-specifically purified protein of similar size to GyrA.

Next, we further optimized the HNG protein-to-Cys-DOPA_5_ molar ratio (Figure 4c,d). HNG-to-Cys-DOPA_5_ ratios of 1:1 to 1:2 were examined, and the ligated peptide His-NKC-Cys-DOPA_5_ was analyzed using SDS-PAGE stained with Coomassie Blue and nitroblue tetrazolium (NBT) (Figure 4c). Under basic conditions, catechols undergo spontaneous oxidation to their quinone form; hence, the NBT staining intensity is directly proportional to the amount of DOPA in the ligation product [42]. The NBT staining intensity was the highest for the ligation product of 0.5 mM HNG and 1 mM Cys-DOPA_5_. Consistent with the SDS-PAGE results, the ligation product of 0.5 mM HNG and 1 mM Cys-DOPA_5_ showed the highest ligation yield of 88.7%, compared to 75% for the product of 0.2 mM HNG and 0.5 mM Cys-DOPA_5_ (quantified using RP-HPLC) (Figure 4d). Harvey et al. reported a programmed protein assembly system that sequentially adds protein modules using intein-mediated protein ligation [40]. Their optimized condition was 0.1 mM protein-thioester and 0.05 mM N-terminal cysteine, with a ligation yield of 76%. In our study, higher concentrations of substrates were used, and there was no need for N-terminal cysteine activation; therefore, the ligation efficiency was slightly higher. Our results demonstrated that the optimal HNG protein-to-Cys-DOPA_5_ ratio was 1:2, with higher ligation efficiency than that at the 1:1 or 1:5 ratio.

### 2.5. Purification of the Ligated Adhesive Peptide

The ligation product (containing 10.4 mg His-NKC-Cys-DOPA_5_) was subjected to preparative HPLC for final purification; the fractions were analyzed using analytical HPLC. HPLC analysis revealed that the fraction contained 7.1 mg His-NKC-Cys-DOPA_5_; the fraction was buffer-changed to deionized water (DW) and lyophilized. The protein yield of each process is listed in Table 2. For the production of longer peptides (>30-mer), bacterial expression is more economical than SPPS. Additionally, in the case of peptide production using intein-cleavage, it has been reported that it can be produced at least two to three times cheaper than SPSS [43]. Although it has not been precisely compared, adhesive antimicrobial peptides can also be produced inexpensively by the intein-mediated ligation method.

### 2.6. Characterization of His-NKC-Cys-DOPA_5_

**Figure 5:**
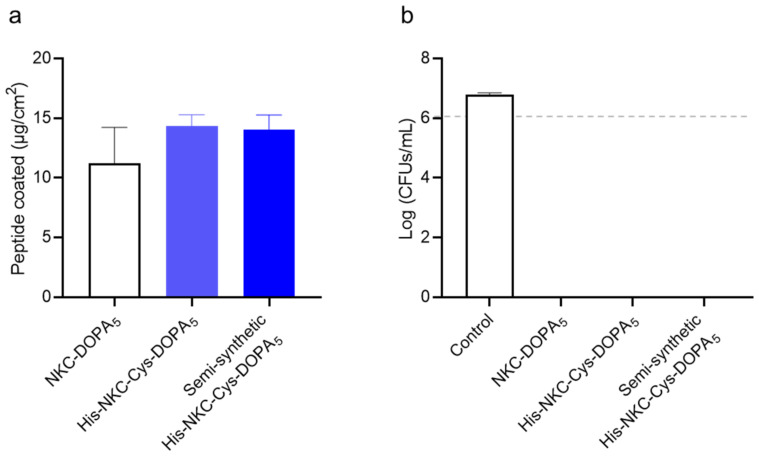
Comparison of the activity of chemically synthesized His-NKC-Cys-DOPA_5_ and semi-synthetic His-NKC-Cys-DOPA_5_. (**a**) Surface-binding activity of peptides. (**b**) Surface antimicrobial activity against *E. coli*. The dashed line indicates the number of cells inoculated. Uncoated wells served as controls. CFUs, colony-forming units.

To assess the functional properties of the semi-synthetic peptide, its adhesiveness and antimicrobial activity on a polystyrene surface were analyzed. The final purified His-NKC-Cys-DOPA_5_ exhibited antimicrobial efficacy against *E. coli*, with an MIC value of 16 μM, the same as that of the peptide obtained by solid-phase peptide synthesis (SPPS). To demonstrate the surface-binding activity, a key feature of the produced peptide, the adhered amount of His-NKC-Cys-DOPA_5_ peptide on a polystyrene 24-well plate was measured (Figure 5a). When the chemically synthesized and semi-synthetic peptides were coated on the polystyrene surface, the amounts adhering to the surface were 14.6 and 14.3 μg/cm^2^, respectively, indicating similar adhesion properties. In addition, the peptide exhibited antibacterial activity after coating (Figure 5b). When *E. coli* was inoculated at 10^6^ cells/mL, surfaces coated with His-NKC-Cys-DOPA_5_ obtained by both chemical synthesis and semi-synthesis completely inhibited bacterial growth. These results were consistent with those of a previous study [35] and demonstrated the feasibility of the intein-mediated protein ligation system for producing UAA-incorporated AMPs with an activity identical to that of chemically synthesized peptides.

## 3. Materials and Methods

### 3.1. Peptide Synthesis

A short peptide containing one cysteine and five DOPA residues (Cys-DOPA_5_) and other standard peptides were chemically synthesized with >90% purity using SPPS at AnyGen Co., Ltd. (Gwangju, Republic of Korea). RP-HPLC (20A gradient system, Shimadzu, Kyoto, Japan) was used to purify the peptides, and an SPD-20A UV-Vis detector was used to collect analyte data at a wavelength of 230 nm. Shimadzu C_18_ preparative (10 μm, 2.5 × 25 cm) and analytical (5 μm, 0.46 cm × 25 cm) columns were used for chromatographic separation using a 1%/min linear gradient of buffer B (0.1% trifluoroacetic acid [TFA] in acetonitrile) in buffer A (0.1% TFA in H_2_O) over a 40 min period at flow rates of 1 and 8 mL/min, respectively. Mass spectrometry was used for the identification of the peptides. All peptides were lyophilized and stored at –20 °C.

### 3.2. Bacterial Strains, Vectors, and Reagents

*E. coli* strains DH5α and BL21 (DE3) (RBC Bioscience, Taipei, Taiwan) were used as hosts for cloning and HNG fusion protein expression, respectively. pET21b (Novagen, Madison, WI, USA) was used to construct expression plasmids. The restriction enzymes *Nde*I and *Xho*I were acquired from New England BioLabs (Beverly, MA, USA), and DNA polymerase and T4 DNA ligase were obtained from TaKaRa Bio (Kyoto, Japan). The oligonucleotides used for gene synthesis and amplification were synthesized at Macrogen (Seoul, Republic of Korea). Tryptone and yeast extract were obtained from Becton Dickinson (Sunnyvale, CA, USA). All other materials were obtained from Sigma-Aldrich (St. Louis, MO, USA), unless otherwise specified.

### 3.3. Construction of Expression Plasmids

DNA fragments encoding HNG protein were amplified via polymerase chain reaction (PCR) from synthetic oligonucleotide and *gyrA* on pTWIN1 (New England Biolabs). The PCR cycling conditions were as follows: 5 min at 94 °C, 30 cycles of 10 s at 98 °C for denaturation, 5 s at 56 °C for annealing, 5 s at 72 °C for extension, and 5 min at 72 °C for the final extension. PCR fragments were separated using 1% agarose gel electrophoresis and purified using a QIAquick Gel Extraction Kit (Qiagen, Hilden, Germany). Amplified fragments were digested with restriction enzymes *Nde*I and *Xho*I and inserted into *Nde*I- and *Xho*I-digested plasmid pET21b, yielding pET21b-HNG. The ligation mixture was transformed into *E. coli* DH5α for plasmid amplification and sequencing. The expression plasmid, pET21b-HNG, was transformed into *E. coli* BL21 (DE3) for HNG expression. The transformed cells were screened on a lysogeny broth (LB; 10 g/L tryptone, 5 g/L yeast extract, and 5 g/L NaCl) agar plate supplemented with 100 µg/mL ampicillin.

### 3.4. Expression and Purification of HNG Fusion Protein

*E. coli* BL21(DE3) cells harboring pET21b-HNG were cultivated in 3 mL of LB containing 100 µg/mL ampicillin at 37 °C under shaking (200 rpm). A 1% (*v*/*v*) overnight culture was inoculated into 50 mL of fresh LB supplemented with 100 µg/mL ampicillin and grown to an OD_600_ of 0.4–0.6. The culture was induced with 0.1 mM IPTG (Bioshop, Burlington, Canada) and grown for an additional 16 h at 25 °C and 200 rpm. Then, the cells were pelleted by centrifugation at 4000 rpm and 4 °C for 20 min. The supernatant was decanted, and the pelleted cells were resuspended in lysis buffer (50 mM Tris-HCl buffer and 300 mM NaCl, pH 8.5). To isolate the HNG fusion protein, the cells were lysed using a tip sonicator (Sonics Vibra-Cell, VCX 750; Sonics & Materials, Inc., Newtown, CT, USA) at 30% amplitude with alternating pulses (5 s on and 5 s off) for 5 min twice. The cell lysate was centrifuged (12,000 rpm, 4 °C, 15 min), and the supernatant was filter-sterilized through a 0.22-μm PES membrane (Stericup Millipore Express Plus; Merck Millipore, Burlington, MA, USA) for subsequent HNG fusion protein purification.

The HNG fusion protein was purified using the ÄKTA Pure fast protein liquid chromatography (FPLC) system (Cytiva Life Sciences, Marlborough, MA, USA) based on nickel-charged immobilized metal affinity chromatography. The clarified cell lysate was loaded onto a pre-equilibrated 5-mL HisTrap FF column (Cytiva Life Sciences) with buffer A (50 mM Tris-HCl and 300 mM NaCl, pH 8.5). After washing with five column volumes (CVs) of buffer A, the bound proteins were eluted using buffers containing increasing imidazole concentrations. Step elution was performed with 5% and 10% buffer B (50 mM Tris-HCl, 300 mM NaCl, and 500 mM imidazole, pH 8.5) over 10 CVs to remove contaminating proteins, and 50% and 100% buffer B over 10 CVs to elute the HNG protein. The protein fraction content was analyzed using SDS-PAGE.

### 3.5. Mass Production of HNG Protein Using Fed-Batch Fermentation

For the large-scale production of the HNG protein, fed-batch fermentation was conducted in 500 L fermenters using R/2 medium, which contains 20 g/L glucose, 0.7 g/L magnesium sulfate heptahydrate (MgSO_4_·7H_2_O), 3 g/L yeast extract, 6.75 g/L potassium dihydrogen phosphate (KH_2_PO_4_), 2.0 g/L ammonium phosphate dibasic ((NH_4_)_2_HPO_4_), 0.85 g/L citric acid, and 5 mL/L of trace metal solution (TMS; 10 g/L FeSO_4_·7H_2_O, 2.25 g/L ZnSO_4_·7H_2_O, 1 g/L CuSO_4_·5H_2_O, 0.5 g/L MnSO_4_·5H_2_O, 0.23 g/L Na_2_B_4_O_7_·10H_2_O, 2 g/L CaCl_2_·2H_2_O, and 0.1 g/L (NH_4_)_6_MO_7_O_24_) [44]. TMS was sterile-filtered and aseptically added to sterile R/2 medium. Glucose and MgSO_4_·7H_2_O were sterilized separately from the culture medium. The pH of the medium was adjusted to 6.8 using 40% (*v*/*v*) NaOH. As the seed culture, recombinant *E. coli* BL21 (DE3) harboring pET21b-HNG was incubated in a 250 mL flask containing 50 mL of LB supplemented with 100 mg/L ampicillin at 37 °C, 180 rpm, for 10 h, and the culture was transferred to a 2 L flask containing 400 mL of LB supplemented with 100 mg/L ampicillin. Thereafter, the 400 mL culture as a seed and 100 mg/L of sterile ampicillin stock were inoculated into 20 L of R/2 medium in a 50 L fermenter. Subsequently, 20 L seed cultures were grown at 37 °C and 150 rpm for 9 h. The OD_600_ was recorded, and the seed culture was aseptically transferred through quick connections to a 500 L fermenter containing 180 L of R/2 medium. For plasmid maintenance, 100 mg/L ampicillin was added to the bioreactor during inoculation. The fermentation parameters, including pH, temperature, agitation, and dissolved oxygen, were monitored and adjusted by a computer-based controlling system (BioCNS, Daejeon, Republic of Korea). To maintain a pH of 6.8, a 25–30% (*v*/*v*) ammonia solution was injected automatically when the pH dropped below 6.5. Dissolved oxygen was maintained at >20% by increasing the agitation speed from 100 to 300 rpm and the aeration rate from 0.75 to 2 vvm. Continuous feeding (500 g/L glucose, 20 g/L MgSO_4_·7H_2_O, and 75 g/L yeast extract) was employed with flow rates manually adjusted as required. The temperature was maintained at 37 °C during the preinduction phase and reduced to 25 °C when the cell density reached an OD_600_ of 60. Once the temperature was stable, 300 mL of 0.1 M IPTG was added to induce protein expression. A sterile antifoaming agent was manually applied. Culture samples were collected periodically for expression analysis. After the maximum OD_600_ was reached, all culture pellets were collected and lyophilized.

To extract HNG protein, the lyophilized cells were resuspended in lysis buffer (50 mM Tris-HCl buffer and 300 mM NaCl, pH 8.5) and lysed using a nano disperser (ISA–NLM100; Ilshin Autoclave Co. Ltd., Daejeon, Republic of Korea) at a rate of 100 mL/min under 1000–1100 bars with three passes. The cell lysate was centrifuged (12,000 rpm, 4 °C, 15 min), and the supernatant was filter-sterilized through a 0.22-μm PES membrane (Stericup Millipore Express Plus) for HNG fusion protein purification. HNG fusion protein was purified using a pre-equilibrated 50 mL Ni Sepharose 6 Fast Flow column (Cytiva Life Sciences) on an ÄKTA Pure FPLC system (Cytiva Life Sciences). The experimental conditions were the same as mentioned above. To remove excess imidazole and NaCl, the purified protein fractions were buffer-exchanged into Tris buffer (50 mM Tris-HCl and 150 mM NaCl, pH 8.5) and concentrated using a VivaFlow 200 tangential filtration system with a 5-kDa cut-off PES membrane (Sartorius, Göttingen, Germany) to recover the flow until the dead volume was reached. After filtration, the concentrate was filtered through a 0.22 μm PES membrane and further concentrated using Amicon Ultra-15 centrifugal filters with a 10-kDa cut-off (Merck Millipore).

### 3.6. Intein-Mediated Cleavage and Ligation

To induce cleavage between the NKC peptide and GyrA intein, HNG fusion proteins (in 50 mM Tris-HCl and 150 mM NaCl, pH 8.5) were incubated with 100 mM MESNA at 25 °C for 24 h. The cleavage product was analyzed using SDS-PAGE and Western blotting. For one-pot intein cleavage and ligation reaction, HNG fusion proteins were incubated in ligation buffer (50 mM Tris-HCl, 150 mM NaCl, 100 mM MESNA, and 10 mM TCEP, pH 8.5) with 0.2–1 mM Cys-DOPA_5_, and the ligation reaction was allowed to continue for 24 h at 25 °C. The protein content of the ligation product was analyzed using SDS-PAGE, NBT staining, and RP-HPLC.

### 3.7. SDS-PAGE, Western Blotting, and NBT Staining

Protein samples were mixed with Novex Tricine SDS sample buffer (2×) and boiled at 95 °C for 10 min, and 5 μL of the samples were loaded onto precast Novex 16% Tricine gels (Invitrogen, Carlsbad, CA, USA). Precision Plus Protein™ Dual Xtra Prestained Protein Standard (Bio-Rad Laboratories, Hercules, CA, USA) was used as the molecular weight marker. Electrophoresis was performed at a constant voltage (125 V) for 65 min. Protein bands were developed using Coomassie Blue or NBT staining.

For Western blot analysis, proteins were transferred onto nitrocellulose membranes (iBlot2 Transfer Stacks) and blotted using an iBlot2 transfer system (Invitrogen). The membranes were blocked with phosphate-buffered saline blocking buffer (LI-COR Biosciences, Lincoln, NE, USA) at 25 °C for 1 h and then incubated with an anti-His mouse monoclonal antibody (cat. SAB1305538, Sigma-Aldrich, 1:1000 diluted) at 4 °C for 16 h. After thorough washing with Tris-buffered saline with 0.1% Tween 20 (TBS-T) for 2 h, the membranes were incubated with 1:20,000 IRDye^®^ 680-conjugated goat anti-mouse IgG secondary antibody (LI-COR Biosciences, Lincoln, NE, USA) at 25 °C for 30 min. After thorough washing with TBS-T for 30 min, the membranes were imaged using an Odyssey^®^ Infrared Imaging System (LI-COR Biosciences).

For the specific staining of redox-active DOPA-containing proteins, NBT was used [45]. The proteins were separated using SDS-PAGE and electroblotted onto a nitrocellulose membrane (Thermo Fisher Scientific, Waltham, MA, USA). The membrane was immersed in NBT/glycinate solution (0.6 mg/mL NBT and 2 M potassium glycinate buffer; pH 10) and incubated at 25 °C in the dark for 45 min. DOPA-containing peptides were stained with NBT/glycinate reagent. A blue-purple stain of bands developed, and the membrane was washed with 0.1 M sodium borate solution and DW.

### 3.8. Protein Quantification

The total protein concentration was measured via the Bradford method using Bio-Rad Protein Assay Dye Reagent Concentrate (Bio-Rad Laboratories, Hercules, CA, USA), following the manufacturer’s instructions. The absorbance of the samples at 595 nm was recorded with a microplate reader (Spark 10 M; Tecan Group Ltd., Männedorf, Switzerland). Protein quantification was based on a calibration curve generated using bovine serum albumin as a standard.

The HNG protein concentration was measured using a His-Tag Detection ELISA kit (Cayman Chemical, Ann Arbor, MI, USA), following the manufacturer’s instructions. HNG was quantified indirectly using an enzyme-linked colorimetric reaction based on the competition between HNG protein and a 6× His tracer (6× His coupled to alkaline phosphatase) for a limited number of His-specific monoclonal antibody-binding sites. The intensity of the generated color was determined spectrophotometrically at 412 nm and was proportional to the quantity of tracer attached to the well and inversely proportional to the quantity of HNG in the well.

### 3.9. RP-HPLC

To quantify the ligated peptides, RP-HPLC was performed using a Vydac C18 analytical column (218TP54; 150 mm × 4.6 mm, 5 μm C18 column; Separation Group, Hesperia, CA, USA) on a 1260 Infinity system (Agilent Technology, Santa Clara, CA, USA). TFA (0.05% *v*/*v*) was added to buffer A (DW) and buffer B (acetonitrile). The flow rate was 1 mL/min, and the injection volume was 5 μL. The elution gradient was 13–28% B over 35 min. Absorbance was measured at 210 and 280 nm. Chemically synthesized peptides (purity of >90%) were analyzed as the standard.

The final ligated peptide was purified using preparative RP-HPLC with a Vydac C18 preparative column (218TP1022; 250 mm × 22 mm, 10 μm) on a multiple preparative HPLC system (LC-forte/R; YMC, Kyoto, Japan). UV/Vis detection was performed at 210 and 230/280 nm and a flow rate of 10 mL/min. The gradient elution was set as follows: 0–50 min (20–35% B); 50.01–51 min (35–90% B); 51.01–56 min (90% B); 56.01–65 min (20% B). Fractions were collected, and those containing ligated peptides were lyophilized using a lyophilizer (FD-8512, ilShinBioBase, Dongducheon, Republic of Korea) for 3 d. The condenser temperature was maintained at –85 °C, with a pressure of 5 mTorr. The lyophilized polymers were collected and stored at –80 °C until use.

### 3.10. Antibacterial Activity Assay

The MIC is the lowest concentration of an agent that causes 100% toxicity. The MICs of the peptides were determined against *E. coli* ATCC 27325 following the Clinical and Laboratory Standards Institute guidelines [46], with minor modifications. *E. coli* cells from a single colony subculture were grown in Mueller–Hinton broth (MHB) until the exponential phase was reached (OD_600_ = 0.4–0.6) and then diluted to 10^6^ colony-forming units (CFUs)/mL with fresh MHB. The AMPs were then added to the diluted bacterial culture in each well of a 96-well polypropylene microtiter plate (Corning, NY, USA), with a final peptide concentration in the range of 0.5–64 μM and a final bacterial concentration of 5 × 10^5^ cells/mL. A growth control, comprising bacterial cells in MHB, and negative control (only sterile MHB) were included. The plates were incubated on a shaking incubator (200 rpm) at 37 °C for 18 h, and MIC values were measured using a microplate reader at 600 nm.

### 3.11. Surface-Binding and Antimicrobial Activity Assays

To analyze the surface-binding and antimicrobial activities of the peptides on a surface, 200 μM peptide solution in 10 mM Tris-HCl (pH 8.5) was coated on a 24-well polystyrene plate (200 μL/well) at 37 °C for 10 min as described previously [35]. After the coating procedure, the plates were washed with DW and air-dried. To quantify the immobilized peptides on the polystyrene well surfaces, the concentrations of loaded and unbound peptide solutions were measured using a micro bicinchoninic acid assay kit (Thermo Fisher Scientific, Waltham, MA, USA), following the manufacturer’s instruction. Bovine serum albumin was used as the standard, and the absorbance at 562 nm was recorded using a microplate reader (Tecan Group Ltd., Mannedorf, Sitzerland). Uncoated wells served as negative controls.

The bactericidal activity of the surface-immobilized peptides was assessed using the International Organization for Standardization 22196 method [47], with minor modifications. Briefly, a mid-exponential phase *E. coli* culture in LB was diluted to 10^6^ cells/mL with 500-fold diluted LB. Two hundred microliters of the bacterial suspension was incubated on uncoated and peptide-coated well surfaces at 37 °C under a relative humidity >90% for 2 h. Then, 200 μL of soybean casein digest broth containing lecithin and polyoxyethylene sorbitan monooleate broth (17 g of casein peptone, 3 g of soybean peptone, 5 g of sodium chloride, 2.5 g of disodium hydrogen phosphate, 2.5 g of glucose, 1 g of lecithin, and 7 g of the nonionic surfactant polyoxyethylene sorbitan monooleate per L) was added to recover the viable cells. The serially diluted bacterial suspension was plated for colony counting.

## 4. Conclusions

The global burden of microbial infections is increasing, and with the emergence of antibiotic-resistant bacteria, it has become difficult to treat some infections [48]. Therefore, the effective prevention of infections remains an urgent point of focus. Here, we developed a surface-binding AMP as a potential antimicrobial coating to kill bacteria upon contact. We utilized an intein-mediated protein ligation strategy to fuse UAAs with AMPs to produce adhesive AMPs. The AMP NKC was expressed in *E. coli* as a His-NKC-GyrA intein fusion protein for inducible cleavage activity, whereas the UAA DOPA was chemically synthesized in the form of Cys-DOPA_5_. The His-NKC-thioester was released by a thiol agent and ligated to the Cys-DOPA_5_ peptide via a native chemical ligation reaction. The titer of the semi-synthetic His-NKC-Cys-DOPA_5_ peptide was 45.2 mg/L from 250 L fed-batch fermentation and subsequent intein-mediated semi-synthesis process, and the peptide exhibited biological activity identical to that of the peptide obtained via SPPS. To the best of our knowledge, this study is the first to demonstrate the incorporation of UAAs into AMPs to produce adhesive AMPs using an intein-mediated protein ligation system. The resulting peptide may potentially be used as a practical and functional antimicrobial coating agent.

## Figures and Tables

**Table 1:** Recombinant protein and synthetic peptides used in this study.

Name	Amino Acid Sequence *	MW ** (kDa)	MIC *** (µM)	Reference
His-NKC-GyrA (HNG)	HHHHHHAPKAMKLLKKLLKLQKKGIGGGGSGCITGDALVALPEGESVRIADIVPGARPNSDNAIDLKVLDRHGNPVLADRLFHSGEHPVYTVRTVEGLRVTGTANHPLLCLVDVAGVPTLLWKLIDEIKPGDYAVIQRSAFSVDCAGFARGKPEFAPTTYTVGVPGLVRFLEAHHRDPDAQAIADELTDGRFYYAKVASVTDAGVQPVYSLRVDTADHAFITNGFVSHA	24.597	-	This study
His-NKC-L	HHHHHHAPKAMKLLKKLLKLQKKGIGGGGSG	3.345	16	This study
Cys-DOPA_5_	CYYYYY	1.017	-	This study
His-NKC-Cys-DOPA_5_	HHHHHHAPKAMKLLKKLLKLQKKGIGGGGSGCYYYYY	4.344	16	This study
NKC-DOPA_5_	APKAMKLLKKLLKLQKKGIGGGGSGGGGSYYYYY	3676.3	16	[35]

* Y represents l-DOPA. ** MW, molecular weight. *** MIC, minimal inhibitory concentration of the peptide against *E. coli*.

**Table 2:** Protein yields during the reaction process.

Procedure	Total Protein	HNG Protein	His-NKC-Cys-DOPA_5_ Peptide
High-pressure homogenization	15.6 g/L	985.4 mg/L (40.1 µM)	
His-tag purification and ultrafiltration	0.77 g/L	483.6 mg/L (19.7 µM)	
Ligation			66.4 mg/L (15.4 µM)
Preparative purification			45.2 mg/L (10.5 µM)

## Data Availability

The datasets generated and/or analyzed during the current study are available from the corresponding author upon reasonable request.
